# Clinicopathological characteristics of Non-Small Cell Lung Cancer (NSCLC) patients with c-MET exon 14 skipping mutation, MET overexpression and amplification

**DOI:** 10.1186/s12890-023-02482-9

**Published:** 2023-07-03

**Authors:** Caixia Ding, Yanyi Qiu, Juan Zhang, Wei Wei, Hongbian Gao, Yong Yuan, Xiaomin Wang

**Affiliations:** 1 Department of Pathology, Shaanxi Cancer Hospital, 309 Yanta West Road, Xi’an, Shaanxi 710000 China; 2 Graduate School, China Pharmaceutical University, 639 Longmian Avenue, Jiangning District, Nanjing, Jiangsu 211122 China

## Abstract

**Purpose:**

MET exon 14 skipping is one of the rare mutations in non-small cell lung cancer (NSCLC), involving its pathogenesis and progression. The performances of several MET inhibitors in clinical trials have been validated based on NGS, immunohistochemistry (IHC), and gene copy number assessments. Thus, a detailed understanding of the relationship between these markers and prognosis is required.

**Methods:**

This study has recruited patients (n = 17) with MET exon 14 skipping mutation and initially screened genes (n = 10) by polymerase chain reaction (PCR) from 257 specimens of NSCLC, including small biopsies and surgical resection. Further, the IHC analysis detected MET overexpression and recorded the score using the MetMAb trial (rial ( recruited patients (n = 17) with MET exstainings). Finally, the fluorescence in situ hybridization (FISH) resulted in the MET amplification with a MET copy number initially screened genes (n = 10) by p.

**Results:**

PCR results indicated strong MET staining ( 3+) in more than 50% of tumor cells. Among the recruited 17 cases of MET exon 14 skipping, 9 cases presented MET amplification, and 10 cases with MET overexpression. These attributes were not correlated to the clinicopathological characteristics and overall survival. In addition, 4 cases showed gene amplification, and 3 cases presented polyploidy condition. The correlation analysis showed a significant relationship between MET amplification and MET overexpression (Pearson’s r2 = 0.4657, P < 0.005).

**Conclusion:**

Together, these findings indicated a significant correlation between MET overexpression and MET amplification in NSCLC patients but no correlation to prognosis.

**Supplementary Information:**

The online version contains supplementary material available at 10.1186/s12890-023-02482-9.

## Introduction

Indeed, the MET proto-oncogene located in the 7q31 locus of chromosome 7 encodes the receptor tyrosine kinase (RTK) enzyme. The binding to a ligand, i.e., hepatocyte growth factor (HGF), often induces its downstream signaling. The regulated expression of the MET proto-oncogene is often related to embryogenesis, wound healing, liver regeneration, angiogenesis, and immunomodulation in diverse physiological processes. Nevertheless, the aberrant MET signaling drives the cells toward tumor development through uncontrolled cell proliferation, survival, invasion, and metastasis, as well as angiogenesis [1]. In this vein, many aberrant activations of the MET pathway have been determined in the tumor tissue, including MET gene amplification, point mutations, gene fusions, exon 14 skipping mutations, protein overexpression, and other activating mutations. Among these MET aberrations, MET exon 14 skipping is one of the rare mutations in non-small cell lung cancer (NSCLC), accounting for 2–4% of lung adenocarcinoma pathogenesis and progression [2]. Due to these facts, MET has become the primary target, in which the development of MET-targeted therapy was approved in 2020 and written in the NCCN guidelines [3]. Accordingly, aberrant MET activation is almost invariably associated with shorter survival and poor prognosis [3, 4].

Over the past decade, several reports indicated the development of various therapeutic strategies addressing the MET exon 14 skipping. In a case, it was reported approximately 5% of NSCLC cases upon progression to epidermal growth factor receptor (EGFR) tyrosine kinase inhibitors (TKIs) and approximately 10% of patients treated with osimertinib (a mutant-selective EGFR TKI) for EGFR T790M-positive NSCLC. Nonetheless, some patients resulted in genetic alteration in the MET receptor, leading to amplified levels of MET proteins and/or the MET gene and resulting in the catastrophe of osimertinib actions. In another case, MET amplification exhibited resistance to ALK inhibitors in the ALK-rearranged NSCLC. In addition, some studies based on MET expression failed to show their potential in HGF/MET targeted therapies. Despite the detection of overexpression, the predominant reason could be the lack of standardization of methodologies, specifically in the development of the scoring systems applied in immunohistochemistry (IHC) determinations or the absence of oncogenic addiction of cancer cells to the MET pathway. Nevertheless, determining the amplification and mutation aberrations often suffer from these pitfalls [5]. In recent times, several advances in developing innovative biomarkers of MET dysregulation in cancer have garnered enormous interest in ascertaining MET-dependent malignancies and developing effective treatment options [6]. Savannah and colleagues demonstrated that MET overexpression and/or amplification was one of the most common resistance mechanisms to osimertinib in 2022 WCLC. Nevertheless, MET detection currently has not reached any clear consensus. Moreover, there still exist a lot of disagreements regarding the relationship between MET exon 14 skipping mutation and gene amplification, as well as protein overexpression.

Inspired by these aspects, in this study, we have recruited NSCLC patients (n = 17) with MET exon 14 skipping mutation in our hospital to conduct relevant clinicopathological analysis. In addition, gene amplification and MET protein overexpression in cases with MET exon 14 skipping mutation were tested to substantially explore the relationship and provide ideas for clinical diagnosis and treatment.

## Materials and methods

### Subjects

In this study, the patient recruitment was performed following the criteria, i.e., the patient with non-small cell lung carcinoma (NSCLC) histology and availability of sufficient cancerous tissues for the extensive studies. Following the criteria, 17 patients with MET exon 14 skipping mutation tumor specimens were included from the total of 257 patients diagnosed with NSCLC at our hospital between 2019 and 2022. Further, IHC and fluorescence in situ hybridization (FISH) validations of C-MET were performed to detect the correlation of MET exon 14 skipping mutations in the recruited cases. Then, the collected tissues, either from surgical resections or core-needle biopsies from the recruited subjects, were used for further analyses. To establish a correlation, the clinical data were extracted from the medical records, including age, sex, tumor disease stage, and clinical follow-up information, among others.

### IHC and scoring

Initially, the IHC evaluations were performed using anti-total c-MET (SP44, Ventana Medical Systems, Roche) rabbit monoclonal antibody as a primary antibody and revealed using an ultraView Universal DAB Detection Kit (760 − 500, Ventana Medical Systems). The IHC staining procedure was carried out according to the manufacturer’s instructions using the Discovery ULTRA platform (Ventana BenchMark Ultra). Briefly, the primary antibody was first incubated for 16 min, and the antigen treatment was performed using the repair solution (ULTRA CC1 95 was carried out accordinthe IHC staining was evaluated by three pathologists according to the MetMab criteria. Accordingly, the high MET for cases was categorized due to the strong MET staining (2 + or 3+), which was presented in more than 50% of tumor cells. In contrast, some cases were categorized as low MET as they showed no such criteria. The MET staining pattern, i.e., predominantly membranous vs. cytoplasmic, was assessed as described elsewhere. This systematic pattern was applied to evaluate both proportion and staining intensity: 0 referred to as no staining or < 50% tumor cells with any intensity; 1 + denoted as ≥ 50% of tumor cells staining with weak or higher staining but < 50% with moderate or higher strong intensity; 2 + indicated as ≥ 50% of tumor cells with moderate or higher staining but < 50% with strong intensity; and 3 + referred to as ≥ 50% of tumor cells staining with strong intensity. Accordingly, the samples that exhibited 2 + or 3 + immunostaining were considered positive for MET overexpression.

### FISH analysis

The FISH evaluation of MET was performed using the unstained, formalin-fixed, and paraffin-embedded (FFPE) tumor tissue sections, as previously described. The FISH analysis was performed using a MET/CEP7 (7q31) probe cocktail (#202,205,001, Guangzhou LBP Medicine Science & Technology Co., Ltd., Guangzhou, China, supplementary Fig. 1 ), according to the manufacturer’s instructions. It should be noted that a minimum of fifty non-overlapping cells with hybridization signals were scrutinized in each sample under a BX51 fluorescence microscope (Olympus, Tokyo, Japan).

Further, the MET amplification was recorded by estimating the ratio between the number of MET copies and the copies of the chromosome 7 centromere (CEP7) using the FISH method. The mean cMET/CEN7 ratio was calculated for each probe by scoring the number of c-MET and CEN7 each nucleus independently. The attributes of MET copy number ≥ 5 and/or MET:CEP7 signal ratio ≥ 2 were denoted as amplification. In contrast, the MET:CEP7 ratio of < 2 was classified as MET non-amplification. While patients with MET copy numbers of ≥ 5 but a ratio of < 2 were classified as MET gain, and both MET and CEP7 copy numbers of ≥ 3 but < 5 were classified as MET polysomy.

### Statistical analysis

Statistical analyses were performed using GraphPad Prism 9.0. Comparing categorical variables was done using the Fisher’s exact test. Using either the Mann-Whitney U test, continuous variables expressing mean ± SD were compared. The correlation of MET amplification and MET overexpression was analyzed using Pearson’s correlation coefficient. Survival curves were generated using Prism. Survival time was regarded as the period from the date of surgery to the date of death of any cause or the date of last follow-up. P < 0.05 was considered significant.

## Results

### Clinicopathological parameters of recruited subjects

The retrospective analysis was performed on the selected cases of NSCLC detected using the ARMS polymerase chain reaction (PCR) from January 2019 to July 2022 in our hospital. Notably, these selected cases of NSCLC included patients with MET exon 14 skipping mutation, 1 case with MET exon 14 skipping mutation combined with KRAS mutation, a case with ALK fusion, and a case with EGFR L858R mutation. Among the total recruited 17 subjects, there were 10 males and 7 females, with a median age of 69 years. The carcinoma status included a case of well-differentiated adenocarcinoma, a case of moderately differentiated adenocarcinoma, and 15 cases of poorly differentiated adenocarcinoma and sarcomatoid carcinoma. Among them, 3 of the recruited cases were in clinical stage I, and 14 were in stage IV (Table 1).

**Table 1:** Clinical characteristics and follow-up of patients (n = 17) carrying MET exon14 skipping mutation

Case	Gender	Age	Specimen	Histological type	TNM stage	Follow up	C- MET D- expression	FISH	Gene test
1	Female	88	Pulmonary puncture	Solid adenocarcinoma	IV	Alive	3+	Cluster amplification	MET exon 14 skipping mutation
2	Male	72	Left upper lobe resection	Lepidic adenocarcinoma 20%, Acinar adenocarcinoma 70%, solid adenocarcinoma 10% (poorly differentiated)	IB	Alive	2+	Cluster amplification	MET exon 14 skipping mutation
3	Male	65	Bronchoscopy	Acinar adenocarcinoma	IV	Dead	Negative	Negative	MET exon 14 skipping mutation
4	Female	50	Left upper lobe resection	20% lepidic adenocarcinoma, 10% acinar, adenocarcinoma 70% solid, adenocarcinoma	IB	Alive	3+	Cluster amplification	MET exon 14 skipping mutation
5	Female	86	Pulmonary puncture	Sarcoma like carcinoma	IB	Dead	Negative	Negative	MET exon 14 skipping mutation
6	Female	56	Left lower lobe resection	Solid adenocarcinoma	IV	Dead (6 m)	2+	Negative	MET exon 14 skipping mutation
7	Male	77	Right lower lobe resection	Solid adenocarcinoma 40%, acinar adenocarcinoma 40%, micropapillary adenocarcinoma 20%	IA	Alive	3+	Cluster amplification	MET exon 14 skipping mutation
8	Female	74	Left lung puncture	Sarcoma like carcinoma	IV	Lost	3+	Cluster amplification	MET exon 14 skipping mutation
9	Male	66	Left upper lobe puncture	Solid adenocarcinoma	IV	Lost	1+	Negative	MET exon 14 skipping mutation, with KRAS mutation Exon 2
10	Female	64	Left upper lobe puncture	Solid adenocarcinoma	IV	Alive	2+	Negative	MET exon 14 skipping mutation
11	Female	77	Left upper lobe puncture	Sarcoma like carcinoma	IV	Lost	3+	Polyploid	MET exon 14 skipping mutation
12	Male	57	Left upper lobe puncture	Solid adenocarcinoma	IV	Alive	3+	Polyploid	MET exon 14 skipping mutation
13	Male	61	Right upper lobe resection	Solid adenocarcinoma	IVB	Lost	3+	Cluster amplification	MET exon 14 skipping mutation
14	Male	69	Cervical lymph node puncture	Solid adenocarcinoma	IV	Alive	3+	No slides	MET exon 14 skipping mutation with ALK fusion mutation
15	Male	72	Pulmonary puncture	Lepidic adenocarcinoma	IV	Alive	3+	Negative	MET exon 14 skipping mutation
16	Male	68	Cervical lymph node puncture	Solid adenocarcinoma	IV	Alive	3+	Negative	MET exon 14 skipping mutation with EGFR L858R mutation
17	Male	72	Pulmonary lobe resection	Solid adenocarcinoma	no data	Alive	No slides	Polyploid	MET exon 14 skipping mutation

### Histopathological and IHC analyses

**Fig. 1:**
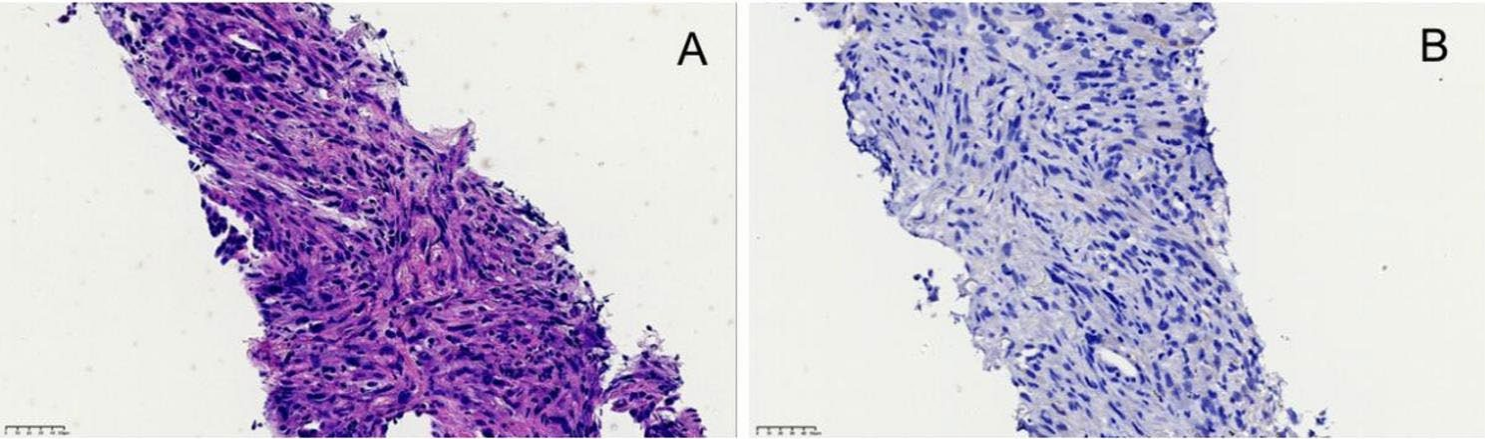
Microscopic images display the sarcomatoid carcinoma showing (**A**) malignant spindle cells and (**B**) negative C-MET, (**A**) x200, and (**B**) x200

**Fig. 2:**
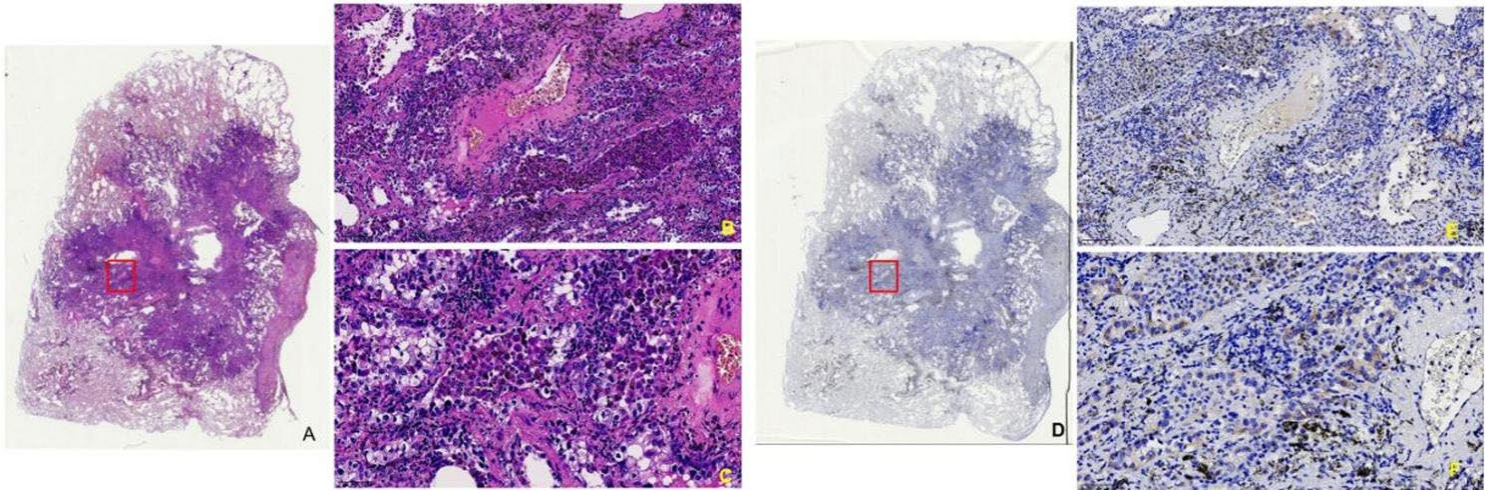
Microscopic images show the (**A-C**) acinar adenocarcinoma characterized by glands that may be oval with tumor cells containing mucin and (**D-F**) focal weakly positive C-MET. (**B**, **E**) x100 and (**C**, **F**) x200

**Fig. 3:**
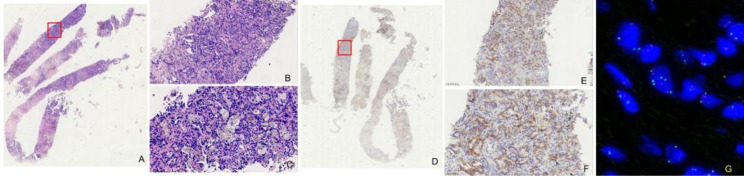
Microscopic images show the (**A-C**) glands in a round to oval with central lumen space surrounded by tumor cells and (**D-F**) moderately positive C-MET. (**B**, **E**) x100 and (**C**, **F**) x200. **(G)** The image shows the corresponding FISH analysis

**Fig. 4:**
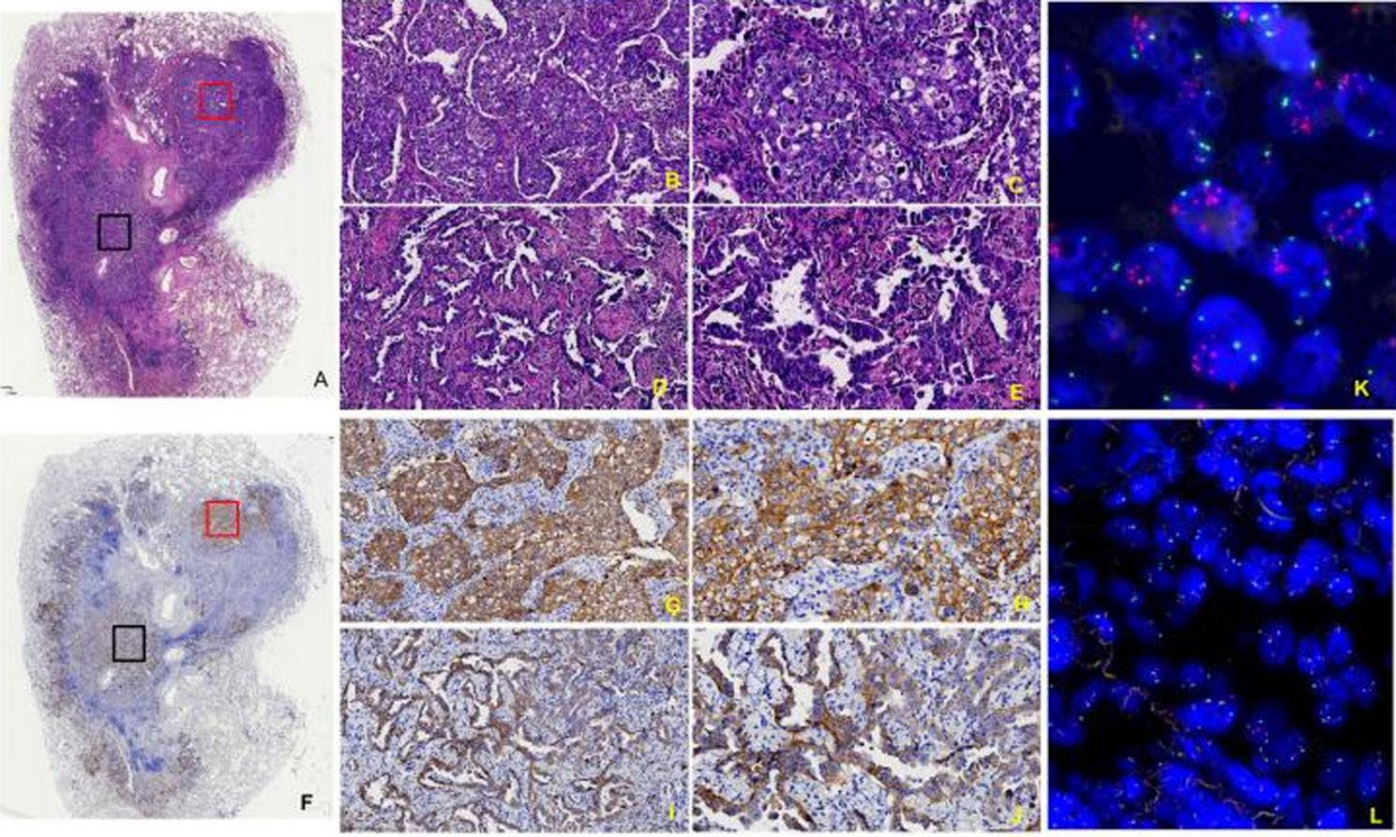
(**A-F)** Images display the acinar adenocarcinoma and solid adenocarcinoma, round to oval glands with polygonal tumor cells growing in sheets and strong positive C-MET. (**B**, **C**- x100 and **D**, **E -** x200, **G**, **H-** x100, and **I**, **J** - x200). **(K, L)** Images indicate the corresponding FISH analysis

In this study, a total of 16 cases/patients with MET exon 14 skipping mutation were included, and their clinicopathological features are listed in Table 1. Further, the microscopic observations were recorded, showing that sarcomatoid carcinoma consisted of malignant spindle cells in the fascicular or storiform patterns (Fig. 1A) and negative C-MET in the patients (Fig. 1B). To this end, the lepidic-predominant adenocarcinoma exhibited bland pneumocytic cells grown along the surface of the alveolar walls in the lungs. The acinar adenocarcinoma was characterized by glands that might be round to oval in shape (Fig. 2A-C**)** or have a more jagged outline with central luminal spaces surrounded by the tumor cells (Fig. 3A-C). The solid adenocarcinoma was characterized by polygonal tumor cells arranged in sheets, lacking recognizable lepidic, acinar, papillary, or micropapillary architectures (Fig. 4A-F).

**Fig. 5:**
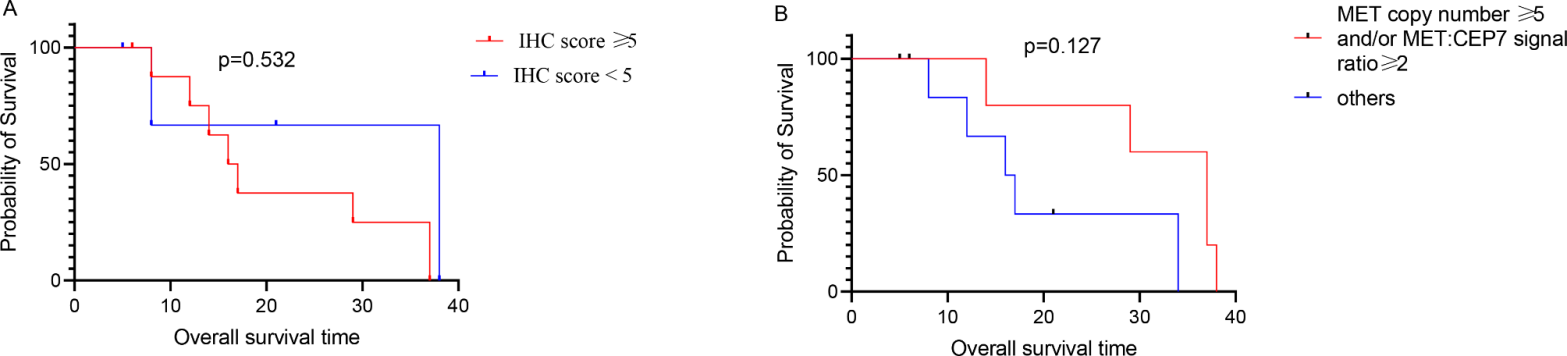
MET Protein Expression and Gene Copy Number vs. Overall survival. **A**. Overall survival probability curves according to MET immunohistochemistry score using. IHC score = 5. **B**. Overall survival probability curves according to MET Fish using MET copy number ≥ 5 and/or MET:CEP7 signal ratio ≥ 2

In IHC staining, the C-MET cytoplasm and membrane staining demonstrated strong positive staining in 10 cases (Fig. 4G and **H**), moderately positive staining in 3 cases (Figs. 3D-F and 4I and **J**), weakly positive in a case (Fig. 2D-F). In contrast, only 2 negative cases were found among the recruited subjects (Fig. 1B). Accordingly, the C-MET expressions of the 16 cases were listed in Table 1. We found no association of MET protein expression and demographic or clinical features, including sex (P = 0.68), stage (P = 0.77), and age (P = 0.67; Table 2). No statistical difference in overall survival (OS) was seen between patients with tumors showing high-MET and low-MET protein expression, as the patients were grouped by IHC score = 5 (IHC score < 5 vs. >=5, P = 0.532, Fig. 5A).

**Table 2:** MET Protein Expression and Gene Copy Number vs. Patient Characteristics

	Met protein expression			MET gene copy		
Characteristics	≥ 2	< 2	P value	≥ 5	< 5	P value
Male/female	7/6	2/1	0.687	3/3	4/6	0.70
Age	68 ± 10.80	72 ± 11.85	0.67	70 ± 13.22	68.30 ± 10.41	0.72
TNM stage						
IV	9	2	0.770	3	8	0.68
I	3	1		2	2	

### MET-FISH analysis

The FISH analysis of 16 samples indicated that the MET cluster amplification in 6 cases (Fig. 4K and **L**), polyploid in 3 cases, and negative cases in 7 cases (Fig. 3G). It should be noted that a case could not be detected due to the lack of a slide. We found no association of MET gene amplification and demographic or clinical features, including sex (P = 0.70), stage (P = 0.68), and age (P = 0.72; Table 2). No statistical difference overall survival (OS) was seen between patients with tumors showing MET amplification and not, as defined by the median (MET copy number ifference overall survival (OS) E2 vs. others P = 0.127, respectively; Fig. 5B).

### Correlation between C-MET IHC and MET FISH

**Fig. 6:**
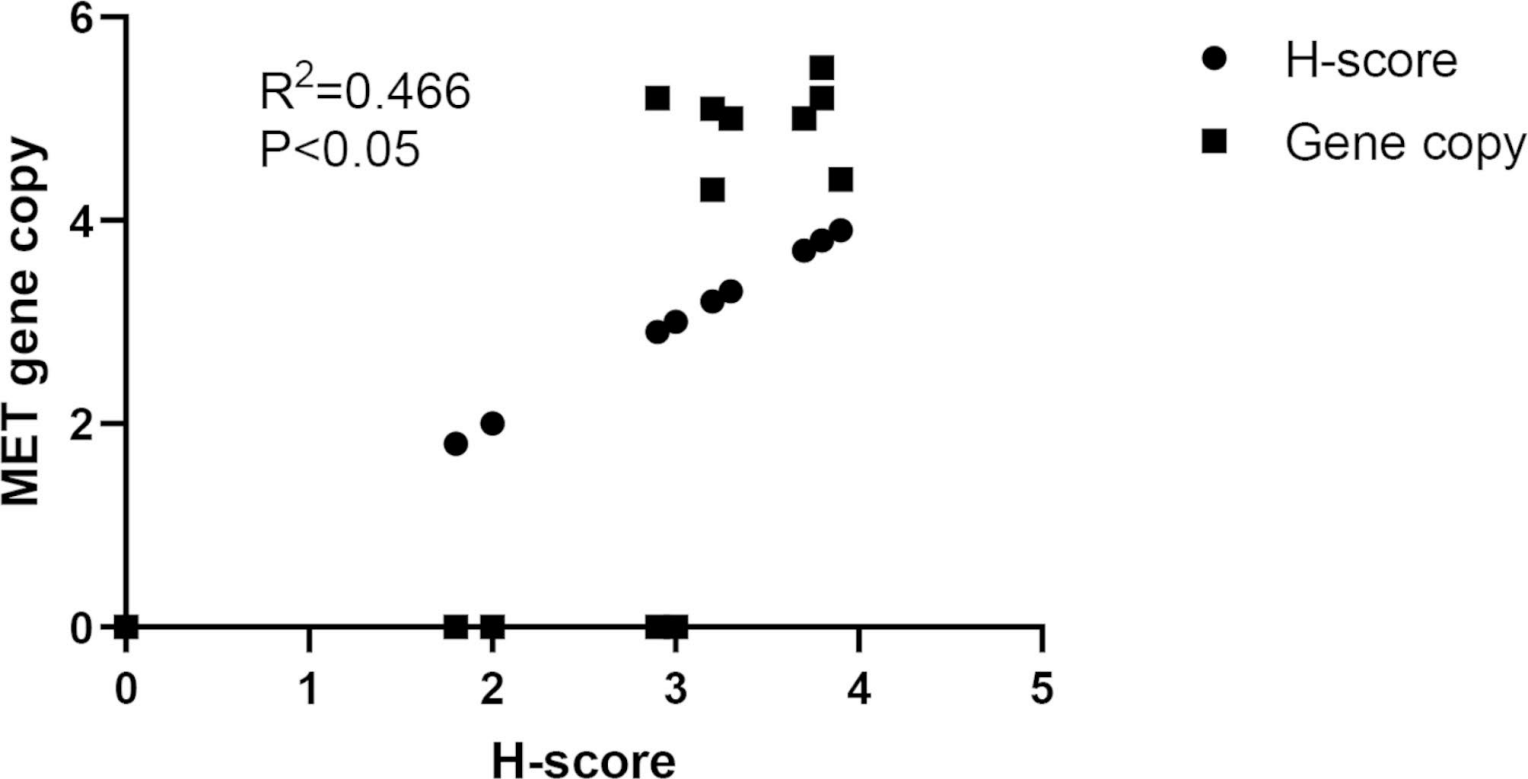
Pearson correlation analysis of MET Protein Expression and Gene Copy Number

Further, the correlation between the C-MET IHC and MET FISH of the selected 16 cases was demonstrated. The C-MET cytoplasm and membrane staining demonstrated strong positive staining in 10 cases and cluster amplification in 3 cases. Further, the FISH analysis indicated polyploid in 3 cases, moderately positive in 3 cases, a case of polyploid, a case of cluster amplification, and a case of negative (Fig. 3G). Pearson’s analysis showed the tight association bewteen MET gene copy number and protein expression (Pearson’s r2 = 0.4657, P < 0.05, Fig. 6).

## Discussion

According to the statistical data, MET exon 14 skipping mutation is evident in about 3% of NSCLC patients, indicating its prognosis and progression [7]. Despite the presence, the pathological type of MET exon 14 skipping mutation is predominantly the below-par differentiated adenocarcinoma. Nevertheless, in some instances, the mutation is combined with MET protein overexpression and MET amplification [8]. In this context, the overexpression of MET is related to the low differentiation efficiency on the histological grade. Notably, the MET exon 14 skipping mutation possessed a higher frequency in pleomorphic carcinoma and adenosquamous carcinoma than in adenocarcinoma [9]. Considering these aspects, the findings in our study are predominantly associated with the solid subtype or sarcomatoid carcinoma of NSCLC.

NSCLC patients with MET exon 14 skipping mutation are often affected in people of an average age of over 70 years or older, dissimilar in cancerous patients with other oncogene-driven forms of NSCLC [10]. Previous reports indicated that NSCLC patients with MET exon 14 skipping mutation often possessed codriver alterations, such as EGFR amplification (6–28%), fibroblast growth factor receptor 1 (FGFR1) alterations (5–17%), KRAS alterations (~ 8%), BRAF alterations (~ 21%), and PIK3CA mutation/amplification (~ 14%) [11].

Notably, the MET expression is heterogeneous. Accordingly, we have observed different levels of molecular heterogeneity, including intra-tumor variability (Fig. 4). These different kinds of histotypes include solid adenocarcinoma and acinar adenocarcinoma. Moreover, the MET IHC showed strongly positive and moderately positive, referring to the cluster amplification in the strongly positive area with FISH analysis. Moreover, the MET status in non-squamous NSCLC tumor areas showed highly heterogeneous among various histotypes, hindering the adequate patient selection for MET-targeted therapies[12]. The intratumor heterogeneity referred to subclonal diversities of tumor cells was observed in this study. The heterogeneity of molecular profiles represented one of the most challenging issues in lung cancer in terms of resultant therapeutic implications [13].

In general, the MET exon 14 skipping mutation may not result in MET overexpression [14]. Accordingly, several reports indicated that MET exon 14 skipping mutations presented no correlation with the MET amplification [15]. However, among 17 cases of MET 14 skipping mutation in our study, MET amplification was observed in 9 cases and overexpression in 10 cases.

IHC staining analysis is often used in clinics due to its feasibility and facile operation. In addition, protein expression levels reflected in the IHC results could be correlated to the amount of gene amplification [16]. Henceforth, we examined the correlation between IHC and gene amplification. It was observed that MET-amplified patients were enriched in patients with positive IHC results. This finding was consistent with the literature demonstrating that high IHC scores correlated with MET gene amplification were measured by the FISH technique. Nonetheless, IHC analysis showed no substantial correlation with the MET exon 14 [17].

In a study of 933 patients with non-squamous NSCLC, the findings displayed that none of the patients with MET exon 14 skipping mutation showed activating mutations in KRAS, EGFR or ERBB2, or rearrangements involving ALK, ROS1, or RET [18]. Contrarily, in another study that included 298 patients with MET exon 14 skipping mutations, KRAS mutation was reported in 3% [19]. The aberrant hyperactivity of the MET receptor caused by the exon 14 skipping mutation seems to have no uniform upregulation in all known downstream effectors, rather gaining a predilection for irrationally activating and consequently relying on the RAS-MAPK pathway [20].

For the treatment of cancers where MET is amplified/overexpressed and activated, MET inhibitors hold tremendous potential. This is crucial since MET amplification, overexpression, and activation in tumor tissue from cancer patients have been linked to poor overall survival. Previously,

NSCLC patients with MET exon 14 mutations and MET amplification had unfavcorable clinical outcome [21, 22]. Our study suggests a possible association between MET amplification and MET overexpression in lung cancer cells, which predicts a poor prognostic course due to MET amplification and overexpression, but the univariate analysis of overall survival in this study did not show significant differences. Future analysis of the association with prognosis should be performed after removal of confounding by multivariable analysis.

The present study does, however, have certain drawbacks, including the low quality of the statistics because of the small sample size. Multivariate analysis could not be applied due to the small number of cases and that analysis with a larger sample size will be necessary in the future. Additionally, the limitation seemed to be due to the primer’s incapacity to detect all known exon 14 skipping events. The probe used in DNA-based methods using amplicons may not sufficiently cover the region of interest.

In conclusion our study analyzed the between MET gene copy number and MET protein expression in 17 NSCLC patients. Among the recruited 17 cases of MET exon 14 skipping, 9 cases presented MET amplification, and 10 cases with MET overexpression. 4 cases showed gene amplification, and 3 cases presented polyploidy condition. A significant relationship between MET amplification and MET overexpression were observed. And yet, no associations of MET copy amplification or overexpression were showed with clinical characteristics and overall survival. Our findings provide baseline biomarker data for MET inhibition-based clinical trials against NSCLC.

## Electronic supplementary material

Below is the link to the electronic supplementary material.

Supplementary Material 1

## Data Availability

The data is available upon reasonable request.Xiaomin Wang should be contacted if someone wants to request the data from the study.
