# Supplementary figures and images for: Clinicopathological characteristics of Non-Small Cell Lung Cancer (NSCLC) patients with c-MET exon 14 skipping mutation, MET overexpression and amplification
Source: BMC Pulm Med. 2023 Jul 3;23:240. doi: 10.1186/s12890-023-02482-9 (PMC10318750; doi:10.1186/s12890-023-02482-9)

*Supplementary figure 1.The ideogram of the FISH MET probe*


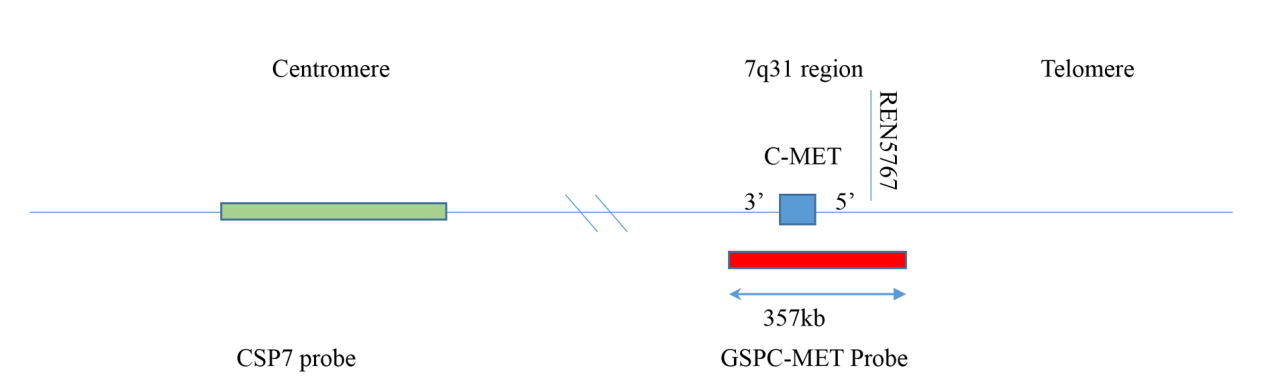

Supplement: Supplementary file 1 — Supplementary Material 1 [file 12890_2023_2482_MOESM1_ESM.docx]
